# Work Intensity and Professional Burden of Psychiatric Nurses

**DOI:** 10.1192/j.eurpsy.2026.10972

**Published:** 2026-07-31

**Authors:** V. Mitikhin, D. Oshevsky, T. Solokhina

**Affiliations:** Mental Health Research Centre, Moscow, Russian Federation

## Abstract

**Introduction:**

Psychiatric nurses perform numerous functions related to the treatment and care of patients, act as a link between doctors and patients, and therefore experience significant professional workload (Yastrebov V.S., Mitina O.A. 2012;112(5):4-12; Tolosa-Merlos D., et.al., 2023: 32(15-16), 5135-5146). The study of the professional burden, that represents the risks associated with exposure to hazardous and harmful factors in the workplace that negatively affect the employee’s life, health, safety and general well-being, will permit to outline ways of it’s prevention.

**Objectives:**

To identify the level of professional burden in psychiatric nurses and to analyze it’s dependence on work intensity.

**Methods:**

The “Questionnaire for assessing the burden of workers in psychiatric institutions” (WHO, 1994) was used. 502 representatives of the nurses staff (61 men male and 441 female women). The average age of respondents was 44.99±11.18 years. The participants were divided into two groups depending on the intensity of work: Group 1 – less than 40 hours per week – 269 (53.59%), Group 2 – more than 40 hours per week – 233 (46.41%). The obtained results were presented as median values with the interquartile range – the first and third quartiles (Me [Q1; Q3]). The Mann-Whitney criterion (U-test) was used for comparison.

**Results:**

The respondents had a medium level of professional burden. Significantly higher values on the integrative scale of the methodology (p=0.045) were found in group 2 – 1.37 [1.23; 1.51] points. In group 1 the values were – 1.32 [1.23; 1.46] points. It was found that the intensity of contact with psychiatric patients was associated with a feeling of physical ill-being. The indicators on the corresponding scale of the questionnaire in group 2 were significantly higher (p=0.005) – 1.33 [1.17; 1.50] in comparison with group 1 – 1.17 [1.17; 1.50] point correspondently. In addition, the socio-psychological burden increased significantly in those working more intensively (p=0.03). The scores on the corresponding scale for nurses in Group 2 were 1.37 [1.25; 1.63] points, while in Group 1 these values were 1.34 [1.20; 1.50] points. It is logical to assume that the mechanisms of forming of physical and socio-psychological stress during long-term and intensive work tend to accumulate, but this position requires additional clarification.

**Conclusions:**

When managing the nurses staff of psychiatric institutions, the intensity of the employees’ work should be taken into account in order to reduce the professional burden.

**Disclosure of Interest:**

None Declared

